# Adaptation of Fig Wasps (Agaodinae) to Their Host Revealed by Large-Scale Transcriptomic Data

**DOI:** 10.3390/insects12090815

**Published:** 2021-09-11

**Authors:** Lianfu Chen, Simon T. Segar, Bhanumas Chantarasuwan, Da-Mien Wong, Rong Wang, Xiaoyong Chen, Hui Yu

**Affiliations:** 1 Key Laboratory of Plant Resource Conservation and Sustainable Utilization, South China Botanical Garden, The Chinese Academy of Sciences, Guangzhou 510650, China; chenllianfu@foxmail.com (L.C.); dmwong87@icloud.cm (D.-M.W.); 2 Southern Marine Science and Engineering Guangdong Laboratory (Guangzhou), Guangzhou 511458, China; 3 Department of Crop and Environment Sciences, Harper Adams University, Newport, Shropshire TF10 8NB, UK; simon.t.segar@gmail.com; 4 Thailand Natural History Museum, National Science Museum, PtthumThani 12120, Thailand; b.chantarasuwan@gmail.com (B.C.); rwang@des.ecnu.edu.cn (R.W.); 5 School of Ecological and Environmental Sciences, Tiantong National Station for Forest Ecosystem Research, East China Normal University, Shanghai 200241, China

**Keywords:** Agaonidae, gene families, pollination ecology, specific mutualism, speciation

## Abstract

**Simple Summary:**

Research on fig wasps has made a considerable contribution to the understanding of insect–plant interactions. However, the molecular mechanisms underlying fig wasp host specificity are poorly understood. This study reports on a relatively large-scale transcriptomic dataset of 25 fig wasp species. We outline potential genetic mechanisms underlying the specific host adaptation by investigating changes in a gene family, in evolutionary rates, and in genes under positive selection. The transcriptome datasets reported here (1) provide new insights into the evolutionary diversification and host specificity of fig wasps and (2) contribute to a growing dataset on fig wasp genomics.

**Abstract:**

Figs and fig wasps are highly species-specific and comprise a model system for studying co-evolution and co-speciation. The evolutionary relationships and molecular adaptations of fig wasps to their fig hosts are poorly understood, and this is in part due to limited sequence data. Here, we present large-scale transcriptomic datasets of 25 fig wasp species with the aim of uncovering the genetic basis for host specificity. Our phylogenetic results support the monophyly of all genera associated with dioecious figs, and two genera associated with monoecious figs, *Eupristina* and *Platyscapa*, were revealed to be close relatives. We identified gene loss and gain, potentially rapidly evolving genes, and genes under positive selection. Potentially functional changes were documented and we hypothesize as to how these may determine host specificity. Overall, our study provides new insights into the evolutionary diversification of fig wasps and contributes to our understanding of adaptation in this group.

## 1. Introduction

Intimate inter-specific interactions are pervasive in nature. Species embedded within these complex networks have consumed each other, provided provisions for each, and competed over ecological and evolutionary time [1].

Evidence for co-evolution in the strict sense is rare [2], but insects and plants clearly form part of each other’s selective landscapes. In some cases, reciprocal selection appears to trigger increased rates of diversification [3]. It is likely that “diffuse” co-evolution acting among groups of individuals is more widespread [4]. In any case, a mechanistic understanding of how selection shapes genomic architecture is hard to achieve in a multi-species setting. Simpler systems that offer a degree of phenotypic matching therefore represent attractive opportunities.

Obligate mutualisms between plants and their pollinators are useful here because the fitness of each partner is closely linked, and trait mismatch or association with an incompatible partner can be expected to result in greatly reduced fitness [5,6,7]. Reciprocal selection likely results in a strong signal, with much of the background ”noise” associated with complex lifecycles involving multiple partners removed.

Here we focus on the interaction between figs and their pollinating wasps. The genus *Ficus* (Moraceae) is pantropical and consists of over 750 species [8]. Each species of fig is pollinated by one to two wasps from the chalcid family Agaonidae. However, as many as nine pollinators can occur across a single host [9]. The inflorescence of a fig tree is known as the ”syconium”; this receptacle has only one entrance (the bract-lined ostiole). Therefore, only specialized fig wasps can enter the syconia to pollinate the female flowers inside (although specialized parasitic fig wasps also exploit this entrance). Once inside, the fig wasp extends its ovipositor into the style of an individual female flower to lay eggs in the ovary within [10,11]. The mutualism between fig and fig wasp has endured for tens of millions of years. Strict mutual adaptation in morphology, behavior, physiology, and development has been documented in this system [12,13].

Olfaction plays an essential role in forming and maintaining the highly specific mutualism between a fig and its corresponding pollinating fig wasps [14,15]. All fig species use chemical cues to attract their specific pollinators, which may include a mixture of compounds or even a single compound, a “private channel” [15,16]. In turn, fig wasps must detect and filter these cues from the surrounding chemical landscape. Besides olfaction, fig wasps can also use short-range tactile and visual cues to determine whether the host is suitable [11,17,18,19]. Detoxification and the immune response of fig wasps also play an essential role in determining host specificity at the larval stage. Fig wasps are also exposed to pathogens including bacteria, fungi, and nematodes and viruses within syconia (often vectored by the wasps themselves). Therefore, fig wasps should be able to defend themselves against pathogens [20,21,22,23].

Adaptive trait matching has been observed between figs and fig wasps [24]. For example, there is a strong correlation between ovipositor length in wasps and style length in figs. It is reported that figs and fig wasps have a consistent relationship of co-cladogenesis and co-evolution with the same subgenus and same section/subsection of figs. Fig sections or subsections are usually pollinated by one corresponding genus of fig wasp [13,25,26,27,28,29]. In total, 19 subgroups of *Ficus* have been described and can be distinguished based on distinct morphological and reproductive characteristics [28,30]. Consequently, it has been predicted that fig wasps are under selection to adapt to changes in their hosts. For example, the thorax, abdomen, and forefoot of fig wasps in the genus *Ceratosolen* all have enlarged spiracles to compensate for the low oxygen environment within the fluid of their hosts’ syconia [30]. In general, wasp heads are flattened and elongated to fit within the narrow bract lining the entrance to otherwise enclosed figs. The arrangement of bracts is also subgenus- or group-specific, are corresponding adaptations are seen in wasps: when the bracts are linear, the head and mandible appendages are longer and thinner, while the pollinators of figs with bracts that are interlocked into a spiral have heads that are flatter, with a soft area for folding, and the mandible appendages are shorter and firmer [18,31]. We suggest that genomic footprints of selection vary among wasps associated with different lineages of figs. Sexual system (monoecy vs. dioecy) is sometimes correlated with other traits in figs and dioecious species tend to be understory specialists. In contrast, pollinators of monoecious figs disperse using above-canopy winds.

Adult female fig wasps are short-lived and non-feeding, and selection should act to favor individuals capable of quickly locating their host fig using species-specific chemical cues from a distance or other visual and tactile signals from a short range. In general, we predicted that these particular organisms would have a reduced genomic architecture to avoid detection of non-target scents. Genes encoding proteins related to feeding, environmental perception, and the immune response would be expressed and/or show signs of positive selection. Variation in the evolutionary rates of genes and gene families were also predicted to be consistent in closely related species and genera of fig wasps when compared to, for example, allo-generics.

Recent studies building on the first fig wasp genome [6] have used an omics approach to greatly enhance our understanding of how selection leaves footprints in expressed genes. For example, reciprocal selection has shaped signal (volatile organic carbon) and receptor (olfactory and gustatory genes) in fig wasps [32,33], while wasps exposed to their host cues actively alter gene regulation of receptors [34]. Here we took a phylogenetically structured approach and compared baseline gene expression in newly emerged adults among (i) a species complex of five pollinating wasps associated with one host (*Valisia*); (ii) one species associated with five hosts (*Blastophaga* sp); (iii) a selection of fig wasps from a single genus spread across several host figs (*Ceratosolen*); (iv) three additional genera sampled for between one to three species; and (iv) the family Agaonidae. Identifying genes capable of species differentiation and evidence for adaptive evolution at the genomic level will assist with understanding the mechanisms shaping reciprocal adaptation, and phylogenetic estimates should be improved through the consideration of many more markers.

Specifically, we used transcriptomic data from newly emerged adult female wasps and performed comparisons among fig wasps and increasingly distant relatives. We addressed the following expectations with reference to the genomes and transcriptomes of one fig wasp (*Ceratosolen solmsi*) and four non-fig wasps (*Apis mellifera*, *Copidosoma floridanus*, *Nasonia vitripennis*, and *Drospophila melanogaster*):

1. In fig wasps, the number of gene contractions in expressed genes is larger than that of expansions due to a reduction in genomic complexity associated with a tight symbiosis;

2. In general, genes under positive selection in fig wasps are mainly related to host location, environmental perception, and the immune response. We expected differences in expression among of genera and species in line with their differing dispersal modes;

3. Fig wasps can quickly adapt to changes of the external environments through gene expression, as evidenced by high turnover in expressed gene families among genera.

## 2. Materials and Methods

### 2.1. Sample Collection

For *de novo* transcriptome sequencing, we sampled a total of 25 taxa of pollinating fig wasps representing the genus *Valisia* (ten species), *Eupristina* (one species), *Platyscapa* (three species), *Blastophaga* (one fig wasp species associated with five fig hosts), *Ceratosolen* (five species), and *Kradibia* (one species) in the family Agaonidae (Hymenoptera) (Table 1). One species, *Ficus hirta*, is pollinated by nine fig wasp species that occupy distinct geographical regions [9]. Eight of these nine fig wasp species share a recent common ancestor. One species, *V. esquirolianae*, enters a close relative of *F. hirta*, *F. triloba*, in certain parts of its range. In this study, we selected four of the eight pollinators *Valisia* sp. 1, sp. 2, sp. 7, and sp. 8, and *V. esquirolianae* as a related species group. In addition, five of the taxa that pollinate *F. pyriformis*, *F. variolosa* and *F. erecta* var. *beecheyana*, *F. formosa*, and *F. abeli* have been identified as a single species by morphology and gene sequencing [35,36,37,38,39]. We considered these to be a monophyletic group.

Fig wasp development is synchronized with that of the syconium, within which they spend most of their lives. Adult female fig wasps are responsible for pollinating and oviposition. We collected syconia close to maturity (wasp emergence). These syconia were cut and placed in nylon bags. Around 50–100 female fig wasps for each species were collected upon emergence and immediately immersed in RNAlater (Ambion/Life Technologies, Thermo Fisher Scientific Inc., Waltham, MA, USA). Samples were stored at −80 °C until RNA was extracted.

### 2.2. mRNA-Seq Library Construction, Illumina Sequencing, Assembly, and Annotation

Total RNA of each species was extracted from 50–100 individuals using a modified CTAB method [40]. The quality of RNA was assessed through gel electrophoresis and using an Eppendorf AG 2231 Bio Photometer Plus (Hamburg, Germany). For each sample, a messenger RNA-Seq library was constructed using an Illumina TruSeq™ RNA Sample Preparation Kit (Illumina, San Diego, CA, USA) following the manufacturer’s recommendations. The isolation of messenger RNA (mRNA), fragment interruption, complementary DNA (cDNA) synthesis, adaptor ligation, PCR amplification, and RNA-Seq were performed by Novogene Bioinformatics Technology Co., Ltd. (Beijing, China). The RNA-Seq library was sequenced on an Illumina Hiseq 4000 platform.

Raw reads were filtered using Trimmomatic v0.38 [41] and adaptors trimmed. Then, the high-quality clean reads were *de novo* assembled to get contigs using Trinity v2.8.5 [42] with default parameters. The expression of the contigs was calculated using RSEM v1.3.1 [43]. TransDecoder v5.5.0 (https://github.com/TransDecoder/; accessed on 9 October 2020) was used to predict the coding sequence (CDS) for each isoform of a gene and the isoform sequence with the highest expression was selected as a unigene. Finally, the protein sequences of all the sampled species were compared with the 5991 Benchmarking Universal Single-Copy Orthologs (BUSCO) in the Hymenoptera_odb10 database to evaluate the integrity of transcriptome using BUSCO v4.1.2 [44] with default settings. The raw sequence data have been deposited in the Genome Sequence Archive (GSA) in the National Genomics Data Center, Chinese Academy of Sciences (https://bigd.big.ac.cn/gsa; accessed on 23 March 2021), under accession number PRJCA004756.

All unigenes were then searched against Nr v20201008 [45], Swiss-Prot v20201011 [46], KOG [47], eggNOG v5.0 [48], and Pfam V33.1 [49] for functional annotation. The gene ontology (GO) annotations were extracted from eggNOG results. The KEGG Automatic Annotation Server (KAAS) with a bidirectional best-hit strategy was used to assign KEGG orthology terms (KO) and to identify the pathways involved [50].

### 2.3. Ortholog Identification and Alignment

To find orthologous genes, CDS and protein sequences of 6 Hymenoptera species and 1 Diptera species, *Drosophila melanogaster*, were collected from NCBI. These species have relatively complete BUSCO and their gene functions have been fully studied. We made a pairwise comparison of the genome or transcriptome protein sequences among these 32 species using the blastp command in diamond v2.0.2.140 [51], and then filtered the blast results using an in-house perl script. Orthologous genes in these filtered data were analyzed using OrthoMCL [52] and clustered with the MCL algorithm [53]. This selection of species includes other Hymenoptera with more complex life histories (including parasitoids and social insects) that also occupy more complex habitats, and they therefore provide a useful baseline for comparison. Based on protein sequence similarity and the mutual best-hit algorithm of all 32 species, orthologous and paralogous gene pairs were identified and clustered into 38,762 orthologous cluster groups (OCGs). Of these, 18,008 OCGs were contained in at least 4 species, and 11,809 OCGs were contained in at least 7 species. These OCGs were selected for codon alignment and downstream analysis.

### 2.4. Phylogenetic Tree and Divergence Time

In total, 661 single/low-copy orthologous genes were found across the 25 species; in 60% of species they were single-copy. The genes with conserved codon sequences of less than 60 bp were filtered by Gblocks v0.91b [54]. The remaining 625 genes were concatenated using an in-house perl script. We estimated the phylogenetic relationships among the species using the maximum likelihood (ML) criterion as implemented in RAxMLv8.2.12 [55].

Two clades in the ML tree, *Valisia* associated with *F. hirta* and *F. triloba* and the five *Blastophaga* taxa, were recovered with relatively low support (90% and 70% respectively). To help resolve these clades, we generated a dataset including only these taxa and recovered 3189 and 5528 single/low-copy orthologous genes with which to generate an ML phylogeny using the above methods. This phylogeny was better supported by and rooted to *Ceratosolen gravelyi* or *Ceratosolen constrictus*. For these two clades, the overlapping 625 single-copy homologous genes were analyzed using the baseml command in PAML v4.9i [56] to generate individual gene phylogenies. These 625 individual phylogenies were integrated into a final phylogenetic tree using an in-house perl script with the branches standardized by the median method.

The divergence time between species was calculated using mcmtree in PAML based on the final topology [56]. The following nodes were calibrated: 127–192 MYA between *Polistes canadensis* and *Apis mellifera*, 172–241 MYA between *Nasonia vitripennis* and *Acromyrmex echinatior*, and 308–366 MYA between *Apis mellifera* and *Drosophila melanogaster*. This was undertaken using constraints derived from “Time Tree” (http://timetree. org/; accessed on 20 October 2020) and the nodes were used to convert relative divergence times to absolute divergence times. Mcmctree was run twice to test convergence (estimates between runs varied by <1%).

### 2.5. Gene Family Expansion and Contraction

In this study, the genes obtained through transcriptome sequencing were all functional genes expressed across study species. We identified gene families using CAFE [57], which employs a random birth and death model to study gene gains and losses in gene families across a user-specified phylogeny. The global parameter λ, which describes both the gene birth (λ) and death (μ = −λ) rate across all branches in the tree for all gene families, was estimated using maximum likelihood. A conditional *p*-value was calculated for each gene family. Only families with conditional *p*-values less than a fixed threshold (0.01) were considered as having an accelerated rate of gain or loss. The expanded and contracted gene families for each branch were tested for enrichment in the GO and KEGG databases. We compared the proportions of enrichment from GO and KEGG between the foreground and background gene families. Significance was tested using Fisher exact tests.

Five species included in our phylogenetic tree had whole genome data available as well as information relating to gene family expansion and contraction [6]. We summarized the number of expanded and contracted gene families expressed for each species in comparison to our phylogenetic tree and assessed how transcriptome data compared to genome level sequences [6]. When considering whole genomes, any change in the quantity of a gene family was regarded as either an expansion or contraction. Here, we used the same standard to summarize the number of such families in the transcriptome data. The correlation coefficient of the number of expansions and contractions and ratio of contractions/expansions between genomic and transcriptomic data were analyzed for each pair using R (version 3.5.2; R Development Core Team, www.R-project.org; accessed on 22 October 2020).

### 2.6. Evolutionary Rate and Positive Selection Analyses

The number of rapid-evolution/positively selected genes detected would have been greatly reduced if only the homologous genes existing in all species or only the conservative blocks in multiple sequence alignments were selected. Therefore, we performed analysis across the genes orthologous in at least four species.

The codeml program in the PAML package [56] with the free ratio model (model = 1) was used to estimate the evolutionary rate following Yang et al. 2014 [58] and Wang et al. 2015 [59]. The lineage-specific mean ratios of non-synonymous (dN) to synonymous (dS) substitutions rates (ω = dN/dS) were estimated for each homologous gene, using the final phylogenetic tree as the guide tree. The values of dN, dS, and dN/dS were obtained for each branch. Assessments of the statistical significance of the differences in the dN/dS ratios along different lineages were conducted using the Wilcoxon rank sum test. To find genes that potentially experienced positive selection, the branch-site model (model = 2 and NSsites = 2) of the PAML package was used, with each branch specified as the foreground branch according to the following rigorous criteria: dN/dS ratio (ω) of the foreground branch greater than the background; *p*-value ≤ 0.05 in the likelihood ratio test [60]; or positively selected sites with a posterior probability greater than 0.95 [61]. The functions of genes with rapidly evolving rates and positive selection were estimated from GO and KEGG. We again compared the proportion of enriched genes from GO and KEGG between the foreground and background gene families. Significance was tested using Fisher’s exact test.

### 2.7. Annotation of Chemosensory Genes

We explored the genetic basis for chemosensory variation among wasps [62]. The number of protein sequences for the following gene families was compared in 25 fig wasps and 7 non-fig wasp insect species: odorant binding proteins (OBPs), olfactory receptors (Ors), chemosensory proteins (CSPs), ionotropic receptors (Irs), and gustatory receptors (Grs). We searched for these families in the Pfam A database using the hmmscan command inHMM v3.3.2, and the results were filtered using a GA bitScore threshold with an e-value of 1e-5 and 25% HMM coverage.

## 3. Results

### 3.1. Comparison of Transcriptome Sequencing and Assembly among 25 Fig Wasp Species

We sequenced transcriptomes of 25 fig wasp species comprising six representative genera of the family Agaonidae (Table 1). For each fig wasp species, we analyzed 20.13–30.62 M (median: 25.04 M) raw read pairs and achieved between 18.93–29.58 M (median: 22.54 M) clean reads after adapter clipping and quality control (Supplementary Materials, Table S1). Among the 25 fig wasp species, only *Valisia* cf. *filippina* had poor transcriptome assembly. Using the Trinity program, next-generation short-read sequences of the other 24 fig wasp species were assembled into 36,024–82,380 transcripts, of which 22,468–44,976 were coded after filtering by TPM expression and ORF search (Supplementary Materials, Table S1). For *Valisia* cf. *filippina*, transcripts and coded transcripts numbered 183,404 and 75,706 respectively, more than two and three times the maximum of 24 fig wasp species. The high-quality transcripts of 24 fig wasp species were subjected to cluster and assembly analyses, resulting in coding of 9579–20,735 unigenes, N50 lengths of 1521– 2728 bp, and GC contents of 36.11–44.91%, while for *Valisia* cf. *filippina* these statistics were 59,860, 774 bp, and 47.1%, respectively. These results indicate that the transcripts assembled for this species were relatively fragmented; for example, a complete sequence obtained in other species would be two fragments in this species. All assembly statistics are summarized in the Supplementary Materials, Table S1.

**Figure 1:**
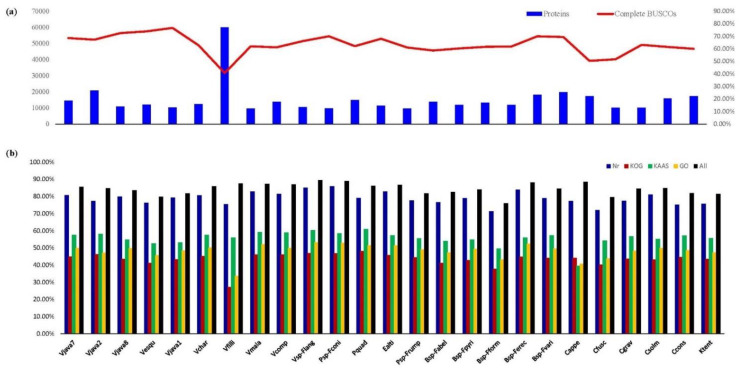
Functional characters of 25 fig wasps~: (**a**) the total number of proteins annotated for 25 fig wasps and the completeness of the transcriptomes identified for them by BUSCO; (**b**) proportions of genes annotated by Nr, COG, KAAS, GO, and all datasets for the 25 fig wasps.

The completeness of the transcriptome was further identified for the 25 fig wasp species using BUSCO analysis (Figure 1). The BUSCO of 24 fig wasp species were all larger than or equal to 50%, while *V.* cf. *filippina* was the lowest at 40.6%. The proportion of unigenes annotated in *V.* cf. *filippina* was 87.44%, which is similar to that of other species studied here (Supplementary Materials, Table S2), indicating that its sequences were normal transcripts. Thus, the 25 fig wasp transcriptomes were all well-assembled and relatively complete.

### 3.2. Unigenes Annotated against Public Databases

Blasting of BLASTP or HMM against protein databases (Nr, Swiss-Prot, KOG, eggNOG, Pfam, KEEG, and GO) revealed that 7859 to 52,340 unigenes (median: 10,437) were successfully annotated for 25 fig wasps (Figure 1; Supplementary Materials, Table S2), and the proportions of unigenes annotated ranged from 75.99% to 89.53% (median: 84. 70%). The proportions annotated against Nr and eggNOG were relatively high, with Nr from 71.45% to 85.89% (median: 78.97%) and eggNOG from 61.03% to 82.72% (median: 76.16%). The proportion annotated against the PFAM was the third highest (from 61.99% to 74.83%; median: 69.14%). The proportions annotated against other databases are given in Table S2. In total, 17,952 and 21,285 OCGs could be annotated by GO and KEGG, respectively, for downstream enrichment analysis.

### 3.3. Phylogenetic Relationships

**Figure 2:**
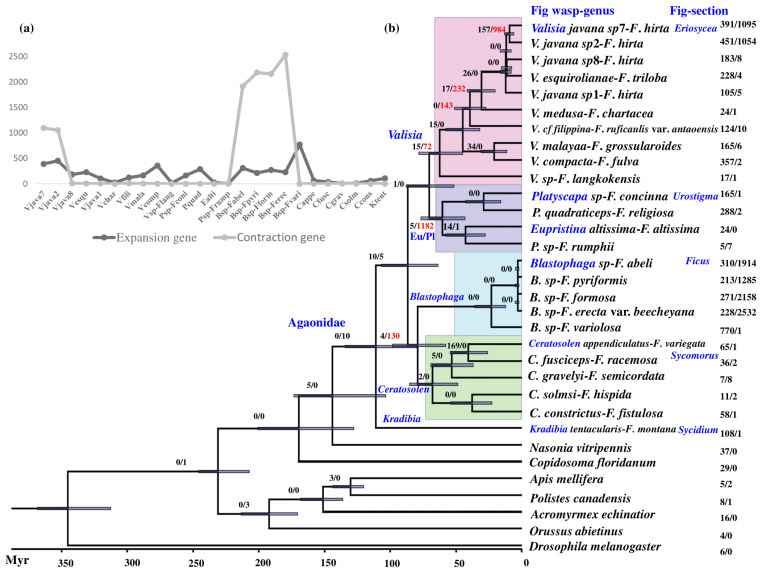
Phylogenetic relationships with gene-family clusters of 25 fig wasps and 7 other insect species: (**a**) comparison of the numbers of expanded (dark line) and contracted (light line) gene families among 25 fig wasps (nodes represent the number of gene families); (**b**) the resultant phylogenetic relationships, including divergence time and the number of expanded/contracted gene families in internal branches and tips (large numbers of contracted gene families are shown in red). Taxonomic information is also given in blue for each clade. Myr means one million years.

The phylogenetic tree generated from 625 single-copy homologous genes was further integrated into one final tree with all branches supported by 100% bootstrap support (Figure 2b). All clades of dioecious fig wasps were monophyletic, while the monoecious fig wasps were polyphyletic, with *Eupristina altissima* and *Platyscapa* sp.*-F. rumphii* branching first and the other two *Platyscapa* species in a different clade. Here, we consider them as one genus clade Eu/Pl.

### 3.4. Gene Family Expansion and Contraction at the Level of Expression

The patterns of expansion and contraction and the ratios of contraction/expansion of the gene family for five insect species, *A. mellifera*, *C. floridanus*, *N. vitripennis*, *C. solmsi*, and *D. melanogaster*, showed no consistent correlation (correlation coefficients (r) of 0, −0.34, and −0.55, respectively; Supplementary Materials, Excel S1). For the genome, the numbers of expanded gene families ranged from 226 to 1426, while for transcriptomes they ranged from 44 to 420; for genome-level family contractions, the range was from 1186 to 2808, while for transcriptomes this number ranged from 365 to 7067. For transcriptomes the number of contracted gene families for all five species was larger than that of the expanded gene family, with the ratio of contraction/expansion of the gene family being between 6.84 and 27.5; this was also the case for genome-level data, which ranged from 1.55 to 12.42, except for one species, *N. vitripennis*, which has a ratio of 0.83.

Compared to the most closely related outgroup taxon, *Nasonia vitripennis*, there are 10 gene families that have undergone contraction in the family Agaonidae. These contracted gene families have functions related mainly to immune response and signal transduction (Table 2; Supplementary Materials, Excel S2). Within the four-genus clade, the numbers of contracted gene families for *Valisia* and Eu/Pl were greater than the numbers that had expanded compared to the neighboring clade (Figure 2). *Valisia* had 15 gene families with evidence of expansion and 72 that had likely contracted. There were no gene families enriched in GO and KEGG for the 72 contracted gene families, but their functions related mostly to signal transduction and genetic information processing (Supplementary Materials, Excel S2). The clade Eu/Pl contained 5 gene families that had expanded and 1182 that had contracted. The functions of gene families enriched in GO (12) and KEGG (8) for the 1182 gene families that had contracted mainly related to environmental information processing. For *Blastophaga* and *Ceratosolen*, there were very few gene family expansions and contractions in a direct comparison, but their ancestral clade contained 4 families that had expanded and 130 that had contracted. For the 130 gene families that had contracted, only 2 of them were enriched in GO and none were enriched in KEGG. The functions of the genes families that had undergone contraction were related to genetic information processing, signal transduction, and energy metabolism (Supplementary Materials, Excel S2).

The numbers of expanded and contracted gene families varied greatly among fig wasp species. This was in contrast to their closest clade with 5–770 (mean ± SE: 184.2 ± 178.8) and 0–2532 (440.0 ± 841.0), respectively (Table 2; Figure 2a,b). While the gene families enriched in GO and KEGG were rarely shared among species, their functions were mainly related to signal transduction, immune system, drug resistance, endocrine, energy metabolism, digestive system, protein production, cytoplasmic translation, and regulation (Supplementary Materials, Excels S3 and S4). The numbers of contracted gene families in genus clades in the phylogenetic tree were greater than that the numbers of expanded genes. The numbers of contracted gene families in most species were lower, except for two related species of *V. javana* sp. 7 and sp. 2 and four taxa of *Blastophaga* (Figure 2a,b). For contracted gene families, the GO- and KEGG-enriched gene families in *V. javana* sp. 7 and sp. 2 were related to amino acid metabolism, signal transduction, energy metabolism or carbohydrate metabolism, and the nervous system, but the specific gene families and metabolic pathways (and, indeed, the proteins that they produce) were different (Supplementary Materials, Excels S3 and S4). Four enzymes or gene families related to protein synthesis (e.g., serine and threonine) enriched in KEGG were shared between *B.* sp.-*F. abeli* and *B.* sp.-*F. pyriformis* (Table 2; Supplementary Materials, Excel S4). Ribosome, valine, leucine, and isoleucine biosynthesis-related genes as well as neurodegenerative disease-related genes were shared between *B.* sp.-*F. formosa* and *B.* sp.-*F. erecta* var. *beecheyana*. No KEGG pathway was shared among the four taxa.

### 3.5. Contraction of Genes Involved in Chemosensory

**Figure 3:**
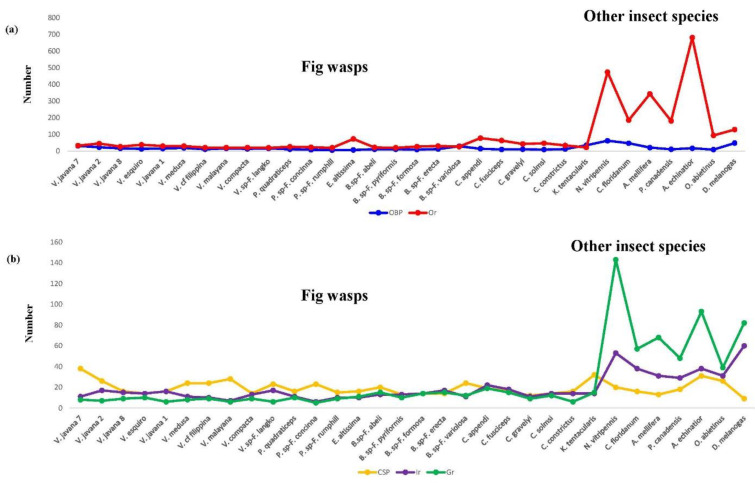
Comparisons of the numbers of genes identified as (**a**) odorant binding proteins (OBPs) and odorant receptors (Ors) and (**b**) as chemosensory proteins (CSPs), ionotropic receptors (Irs), and gustatory receptors (Grs). The numbers of genes in OBP and CSP families in fig wasps were similar to those in other insects, while the numbers of genes in Or, Ir, and Gr gene families were significantly lower in fig wasps.

It has been reported that some chemosensory gene families in fig wasps have experienced dramatic contractions in relation to other insects [6]. Therefore, we compared the numbers of genes in OBP, Or, CSP, Ir, and Gr families among fig wasps and other insect species (Table 3; Figure 3a,b). The numbers for OBPs and CSPs in the fig wasps were 6–35 (14.7 ± 7.4) and 12–38 (19.5 ± 6.6), while in other insects these figures were 9–62 (30.7 ± 21.2) and 9–31 (19 ± 7.5); there were no significant differences in OBP and CSP numbers when comparing fig wasps with other insects (*t*-test: *t* = −1.966, *p* = 0.094; *t* = 0.165, *p* = 0.870). The numbers of genes for Or, Ir and Gr families in fig wasps were 20–78 (33.6 ± 16.4), 6–22 (13.2 ± 3.6), and 5–19 (10.2 ± 3.6), respectively, while those of the other insects were 94–681 (298.3 ± 214.5), 29–60 (40.0 ± 12.0), and 39–143 (57.7 ± 35.1); there were significantly lower numbers of genes in each of these three sensory classes in fig wasps when compared to other insects (*t* = −3.262, *p* = 0.017; *t* = −5.86, *p* = 0.001; *t* = −4.931, *p* = 0.003).

### 3.6. Rapidly Evolving Genes (REGs)

In the family Agaonidae, we detected 857 rapidly evolving genes (REGs) (Table 2; Figure S1b). Of these REGs, 484 and 45 were enriched in GO and KEGG, respectively; their gene functions were related mainly to signal transduction, immune response, and antibacterial systems (Table 2; Supplementary Materials, Excels S5 and S6). For the Eu/Pl, *Ceratosolen*, *Valisia*, and *Blastophaga* genus clade, we detected higher numbers REGs, from 2073 to 2572 (2262.8 ± 222.5), than the numbers of positively selected genes, which ranged from 10 to 85 (62.8 ± 35.4). Low numbers of REGs were enriched in GO in the clades of *Valisia* (17), Eu/PL (14), *Blastophaga* (1), and *Ceratosolen* (4) (Supplementary Materials, Excel S5) while no REGs were enriched in KEGG. There were 13 REGs enriched in GO that were shared between *Valisia* and Eu/Pl, and these mainly related to mitochondrial function. The REGs enriched in GO in *Blastophaga* were related to the vesicle-tethering complex, while four of the *Ceratosolen* REGs encoded antibacterial peptides (Supplementary Materials, Excel S5).

The numbers of REGs were lowest (804–1162) among the five related *V. javana* species (*t* = −4.505; *p* < 0.000) and among the five *Blastophaga* taxa (286–967; *t* = −5.408; *p* < 0.000) (Figure S1a,b; Table 2) in comparison to the remaining species (890–1864). Among these other species, zero to seven REGs were enriched in GO and zero to five were enriched in KEGG. None of the five related *V. javana* species had REGs enriched in GO and only three of them had REGs enriched in KEGG (Supplementary Materials, Excel S5 and S6): the PEP4 gene in *V. javana* sp. 2; CYP6, ydfG, and USO1 in *V. esquirolianae*; and ANK in *V. javana* sp. 1. Among the five taxa of *Blastophaga*, the numbers of GO- and KEGG-enriched genes were 0–193 and 0–20, respectively. Most REGs in *B.* sp.-*F. abeli* and *B.* sp.-*F. pyriformis* enriched in GO/KEGG were related to carbohydrate and energy metabolism, environmental adaptation, the ribosome (genetic information processing), and neurodegenerative disease. In addition, 35 REGs were enriched in GO for *B.* sp.-*F. variolosa* and their functions were related to the development of cells, tissues, and organs.

### 3.7. Positively Selected Genes (PSGs)

In the family Agaonidae, we detected 68 PSGs and none of these were GO- or KEGG-enriched (Table 2; Figure S1b and Excels S7 and S8). These PSGs mainly coded for proteins related to signal transduction (CAMK1, FOXG, GRIN, IRAK4, PLK2, STAT5B, and KCNH8), immune response, antibacterial systems, genetic information processing (transport, translation, transcription, membrane trafficking, replication, and repair), development and regeneration, amino acid metabolism, and energy metabolism (Supplementary Materials, Excel S9). When making comparisons at the genus level, we found 75–110 (87.8 ± 15.4) genes under positive selection; of these genes, 0–13 were enriched in GO and 0–2 were enriched in KEGG (Figure S1b and Excels S7 and S8). These genes mainly coded for proteins involved in energy metabolism, genetic information processing, and environmental adaptation. There was a single common KEGG, genetic information processing, shared among the three genera associated with dioecious hosts.

In total, the numbers of PSGs were lower than those of REGs for Agaonidae, genera, and species (Table 2; Figure S1a,b). In contrast to REGs, the numbers of PSGs were much higher among the five related *Valisia* species (128–207 (*t* = 8.773, *p* < 0.001)) and the five taxa of *Blastophaga* (227–288 (*t* = 15.227, *p* < 0.001)) compared to those of other species (22–96) (Table 2; Figure S1). The number of GO- and KEGG-enriched PSGs in most species was 0 (Supplementary Materials, Excels S7 and S8). Among the five related *Valisia* species, only the PSGs of *V. esquirolianae* were enriched in GO and KEGG which were related to genetic information processing, amide synthesis, and peptide metabolism. There was one common PSG among the five related *Valisia* species, ANK (Supplementary Materials, Excel S9). Among the five taxa of *Blastophaga*, only *B.* sp.-*F. abeli* and *B.* sp.-*F. variolosa* had PSGs enriched in GO (Supplementary Materials, Excel S7 and S8). The GO enriched in *B.* sp.-*F. abeli* were related to biological processes, such as mid-gut development, response to external stimulus, and regulation of localization, while the PSGs of *B.* sp.-*F. variolosa* enriched in GO were mainly related to transcription and regulation of biosynthetic process (Supplementary Materials, Excel S7). There were no common PSG genes among the five related taxa (Supplementary Materials, Excel S9).

## 4. Discussion

### 4.1. Comparisons among Agaonidae

Our results demonstrate a dramatic contraction across a wide range of gene families in pollinating fig wasps compared to other Hymenoptera, offering support for results from previous studies [6,33,63,64]. It has been reported that in the genomes of *Ceratosolen solmi, Eupristina verticillata*, and *Wiebesia pumila*, many gene families related to chemosensory, detoxification, and innate immune response are reduced [6,33,63,64]. This finding is reflected in our transcriptome study: for the whole family of Agaonidae, the contracted genes families at the level of expression in newly emerged adult fig wasps were mainly enriched in relation to signal transduction and immune response in comparison to other parasitic wasps (*Nasonia vitripennis* and *Copidosoma floridanum*), ants, bees, and other Hymenoptera. Genes encoding for proteins used in environmental information processing, antibacterial defenses, amino acid and energy metabolism, cell growth and death, and the nervous system evolve quickly and are under positive selection. In the genome of *Ceratosolen solmi*, 13 genes were identified as rapidly evolving genes and they often act as transporters of signals or substances [6].

Figs provide a relatively safe nursery for developing larvae, shielding them from the external environment and many antagonists [6,10]. Fig wasps display morphological, behavioral and physiological adaptations related to life inside the syconium, where nutrients (starch and amino acid) are produced within endosperm tissue. It is important, however, to note the context of this study. We generated transcriptomes from adult females and did not explicitly account for age or external stimuli; as such, our main findings are relevant to newly emerged wasps only. The inconsistent pattern of gene family expansion and contraction between genomic data and our transcriptomic data also implies that gene expression is easily influenced by the environment, although the number of gene family contractions was typically larger at both levels.

Chemosensory genes play an important role for insects in foraging, ovipositioning, mating, avoiding natural enemies, and searching for hosts [65]. Our results showed that Ors, Grs, and Irs in fig wasp species are reduced to a greater extent in comparison to other insect species, with similar numbers as *Ceratosolen solmi*, *Eupristina verticillate*, and *Wiebes pumila*—Ors: 43–46, 48, and 67; Grs: 5, 4, and 4; Irs: 11–31, 30, and 16, respectively [6,33,63]. Genes in the Or family are related to the detection of volatile signals, while those in the Gr family generally contribute to the detection of soluble chemical substances. Groups of receptors are essential for survival and reproduction, e.g., for host location and avoidance of natural enemies and while searching for mates [66]. In addition to detecting volatile odor molecules, such as ammonia, phenylacetaldehyde, and hexanal, the genes in the Ir family are also involved in taste, temperature, and humidity sensing when used alongside the genes in the Or family [67]. The number of Ir genes in the genome of the monoecious fig pollinator *Eupristina verticillata* was found to be higher than two other fig wasp species whose hosts are dioecious figs. This finding may be related to the adaptation to finding a host at a greater distance [68] (Yang et al. 2015). The significant contraction of Ors, Grs, and Irs in fig wasps suggests that they achieve host specificity through reduced gene content and expression of chemosensory receptor genes at the transcriptome level; this likely allows them to better detect species-specific fig volatiles.

### 4.2. Comparisons at Genus Level

At the genus level, the numbers of contracted gene families were far greater than those of expansions. Moreover, the more phylogenetically distant the genera under comparison were, the greater the number of contracted gene families detected. In contrast to *Valisia* (the pollinator of a dioecious species and a close relative in the current sampling regime), the monoecious Eu/Pl clade had 1182 contracted gene families mainly enriched in “environmental information processing”. We speculate that this was due to the adaptation of pollinators to the monoecious reproduction of their hosts. There were more REGs and PSGs, but their functions were rarely enriched in GO and KEGG, which indicates that the adaptation of these functional genes at the level of the genus was random and scattered. In terms of PSGs, only one enriched pathway of genetic information processing (ko03010) was shared among the three dioecious genera; this is a candidate pathway for adaptation to the dioecious breeding system, but more dioecious genera are needed to test this hypothesis.

### 4.3. Comparisons among Species

Our phylogenetically structured sampling strategy also allowed us to assess differences among species within Agaonidae. The GO- and KEGG-enriched genes across gene families for species may have related to the enclosed nature of the syconia, and functions included signal transduction, immune response, drug resistance, endocrine regulation, energy metabolism, digestion, protein production, cytoplasmic translation, and regulation. However, the exact genes expressed were rarely the same across species, which was consistent with the highly species-specific nature of the interaction with their host. REG and PSG genes among species were mainly related to energy metabolism, drug resistance, environmental information processing, genetic information processing, P450 function, and carbohydrate metabolism. However, few genes could be enriched with GO and KEGG at the species level, a similar result to the genus-level comparisons.

### 4.4. Comparisons among Closely Related Species

Among closely related *Vallisia* species or taxa of *Blastophaga*, the numbers of contracted gene families varied greatly, which may have been due to the low expression of these genes, implying that fig wasps can quickly respond to host changes through gene expression. Future studies focusing on genomic sequencing would provide the context needed to confirm this. Moreover, the KEGG-enriched genes in contracted gene families were rarely shared across close relatives; this indicates differential expression and potentially subtle differences related to host identity. Fig wasps can likely respond quickly to environmental changes through gene expression. Meanwhile, the PSG numbers increased significantly in these comparisons, suggesting that these species/taxa were under selection.

The ANK was the only gene under positive selection that was shared among the five related *Valisia* species, and it was also one of the only rapidly evolving genes enriched in *V. javana* sp 1. The function of ANK relates to the IMD pathway and it can be activated by the infection of Gram-negative bacteria. Activation of ANK subsequently leads to the activation of NF-κB signal transduction pathways, which induce the synthesis of antibacterial peptide genes and other genes involved in the immune response (for example, see [69]). The hosts of the *V. javana* clade are two related species, *F. hirta* and *F. triloba*, in the same *Ficus* subsection as *F. hirta.* These figs have a broad geographical distribution, which implies that their syconia have similar bacterial infections countered by the same adaptation in five pollinator species. Further, the ANK gene is the only gene family found in all currently sequenced bracoviruses (BVs) and ichnoviruses (IVs; [70]) of parasitoid wasps [71], where they are likely used to manipulate host physiology and defense [72].

## 5. Conclusions

Our large-scale transcriptomic dataset was derived from 25 fig wasps, 6 other Hymenoptera species, and 1 Diptera species. First, we reported the genomic mechanisms underlying this highly species-specific interaction at the levels of family, genus, and species. The rapid diversification of fig wasps is related to their symbiosis with highly species-specific fig hosts and the expansion and contraction of gene families, candidate REGs, and PSGs. Differences in gene function across fig wasps may reflect their long-lasting relationships with figs and rapid adaptations in the locations of host syconia. Our dataset will be of interest for studies of the co-evolution, co-speciation, and biodiversity of figs and fig wasps.

## Figures and Tables

**Table 1:** Information on fig wasps used for transcriptome sequencing. *Valisia* sp. 1, sp. 2, sp. 7, and sp. 8 are the different pollinating species with allopatric distribution within a single host, *F. hirta* [9]. It has been reported that the pollinating wasps in *F. pyriformis*, *F. erecta* var. *beecheyana*, *F. formosa*, *F. variolosa*, and *F. abeli* are the same species according to the morphology and DNA sequences [35,36,37,38,39]. Our results also confirmed that they form a single clade. We considered them as one species with five hosts: *B.* sp.-*F. abeli*, *B.* sp.-*F. pyriformis*, *B.* sp.-*F. formosa*, *B.* sp.-*F. erecta* var. *beecheyana*, and *B.* sp.-*F. variolosa*.

Fig Wasp		Host		Sampling Site		
Genus	Species	Species	Section	Site	Latitude	Longitude
*Platyscapa*	*quadraticeps*	*F. religiosa*	*Urotigma*	Myanmar	22.013	96.073
	sp.	*F. concinna*	*Urotigma*	China—Guangdong	23.816	114.013
	sp.	*F. rumphii*	*Urotigma*	Myanmar	21.984	96.060
*Eupristina*	*altissima*	*F. altissima*	*Urotigma*	China—Guangdong	22.763	113.612
*Valisia*	sp. 1	*F. hirta*	*Eriosycea*	China—SCBG	23.179	113.352
	sp. 2	*F. hirta*	*Eriosycea*	Thailand—Chiang Mai	18.809	98.914
	sp. 7	*F. hirta*	*Eriosycea*	Thailand—Chantaburi	12.774	102.096
	sp. 8	*F. hirta*	*Eriosycea*	Thailand—Trang	7.467	99.639
	*esquirolianae*	*F. triloba*	*Eriosycea*	China—Guangdong	23.635	113.770
	*medusa*	*F. chartacea*	*Eriosycea*	Thailand	7.556	99.766
	cf. *filippina*	*F. ruficaulis var. antaoensis*	*Eriosycea*	Taiwan	21.962	120.811
	*malayana*	*F. grossularoides*	*Eriosycea*	Thailand—Narathiwat	5.799	101.762
	*compacta*	*F. fulva*	*Eriosycea*	China—Guangdong	8.776	99.724
	sp.	*F. langkokensis*	*Eriosycea*	China—Guangdong	24.252	112.036
*Blastophaga*	sp.	*F. abeli*	*Ficus*	China—Guangdong	23.636	113.780
	sp.	*F. pyriformis*	*Ficus*	Thailand	18.504	98.665
	sp.	*F. erecta var. beecheyana*	*Ficus*	China—Guangdong	23.761	113.920
	sp.	*F. formosa*	*Ficus*	China—Guangdong	23.623	113.811
	sp.	*F. variolosa*	*Ficus*	China—Guangdong	23.180	113.275
*Ceratosolen*	*appendiculatus*	*F. variegata*	*Sycomorus*	China—Guangdong	23.181	113.359
	*fusciceps*	*F. racemosa*	*Sycomorus*	Myanmar	20.765	96.945
	*gravelyi*	*F. semicordata*	*Sycomorus*	Thailand—Chiang Mai	19.362	98.922
	*constrictus*	*F. fistulosa*	*Sycomorus*	China—Guangdong	23.156	112.511
	*solmi*	*F. hispida*	*Sycomorus*	China—SCBG	23.179	113.352
*Kradibia*	*tentacularis*	*F. montana*	*Sycidium*	Thailand	7.557	99.776

**Table 2:** Numbers of gene family expansions/contractions, rapidly evolving genes (REGs), and positively selected genes (PSGs) and the numbers of genes enriched in GO/KEGG according to family, genus, species, and related species/taxa of fig wasps given in the phylogenetic tree.

Items	Gene Family				REG		PSG	
	Expansion		Contraction					
	Total Number	Number of Gene Families Enriched in	Total Number	Number of Gene Families Enriched in	Total Number	Number of Genes Enriched in	Total Number	Number of Genes Enriched in
		GO/KEGG		GO/KEGG		GO/KEGG		GO/KEGG
Family								
Agaonidae	0	0/0	10	0/2	857	484/45	68	0/0
Genus								
								
Eu/Pl	5	0/0	1182	12/8	2572	14/0	85	13/0
*Ceratosolen*	2	0/0	0	0/0	2073	4/0	81	11/1
*Valisia*	15	0/5	72	0/0	2133	17/0	75	9/2
*Blastophaga*	0	0/0	0	0/0	2273	1/0	110	0/2
Species in Eu/Pl clade								
*Platyscapa quadraticeps*	288	15/0	2	0/0	890	0/5	23	0/0
*P.* sp.-*F. concinna*	165	20/0	1	0/0	1195	1/2	39	0/0
*P.* sp.-*F. rumphill*	5	0/0	7	0/0	1027	0/0	28	5/0
*Eupristina altissima*	24	0/2	0	0/0	1258	2/1	22	0/0
Species in *Ceratosolen*								
*C. appendiculatus*	65	0/14	1	0/0	1088	0/0	32	0/0
*C. fusciceps*	36	2/1	2	0/0	1223	1/2	39	0/0
*C. gravelyi*	7	0/0	8	0/0	1245	0/0	29	0/0
*C. solmsi*	11	0/0	2	0/0	1012	0/0	24	0/0
*C. constrictus*	58	77/2	1	0/0	1144	0/0	27	1/0
Species in *Kradibia*								
*K. tentacularis*	108	88/4	1	0/0	1649	0/0	43	0/0
Species in *Valisia*								
*V. medusa*	24	0/0	1	0/0	1864	4/0	63	0/0
*V.* cf *filippina*	124	31/8	10	0/0	1664	0/0	96	0/0
*V. malayana*	165	2/0	6	0/0	1242	0/0	28	0/0
*V. compacta*	357	75/9	2	0/0	1549	0/0	44	0/0
*V. sp-F. langkokensis*	17	0/1	1	0/0	1773	7/4	91	18/2
Related species of *V. javana*								
*V. javana* sp. 7	391	10/4	1095	257/18	804	0/0	207	0/0
*V. javana* sp. 2	451	79/16	1054	29/5	803	0/2	177	0/0
*V. javana* sp. 8	183	1/0	8	0/0	1065	0/0	128	0/0
*V. javana* sp. 1	105	4/1	5	0/0	1162	0/1	177	0/0
*V. esquirolianae*	228	16/17	4	0/0	1032	0/3	155	18/0
Species in *Blastophaga*								
Related taxa—five taxa of one species in different hosts								
*B.* sp.-*F. abeli*	310	62/1	1914	137/16	533	193/20	275	3/0
*B.* sp.-*F. pyriformis*	213	44/2	2185	329/31	967	112/10	288	0/0
*B.* sp.-*F. formosa*	271	14/3	2158	283/25	286	0/0	227	0/0
*B.* sp.-*F. erecta var. beecheyana*	228	31/12	2532	546/12	324	0/1	230	0/1

Note: The fig wasp species associated with the two monoecious genera, *Eupristina* and *Platyscapa*, converged into one clade in the phylogenetic tree, so we considered them as the clade Eu/Pl. *V. javana* sp. 1, 2, 7, and 8 are related species in the same host with different distributions [9]. *V. esquiroliana* is the pollinator of *F. triloba* which is closely related to *F. hirta*. The five taxa of *Blastophaga* are closely related to each other; indeed, they are likely a single species.

**Table 3:** Numbers of OBP, Or, CSP, Ir, and Gr genes among 25 fig wasps and 7 other insect species.

Species	OBP	Or	CSP	Ir	Gr
*Valisia javana* sp. 7	31	33	38	11	8
*V. javana* sp. 2	23	45	26	17	7
*V. javana* sp. 8	16	27	16	15	9
*V. esquirolianae*	13	38	14	14	10
*V. javana* sp. 1	15	30	16	16	6
*V. medusa*	19	30	24	11	8
*V.* cf *filippina*	11	21	24	10	9
*V. malayana*	16	21	28	7	6
*V. compacta*	13	21	14	13	9
*V.* sp.-*F. langkokensis*	16	20	23	17	6
*Platyscapa quadraticeps*	11	26	16	11	10
*P.* sp.-*F. concinna*	8	23	23	6	5
*P.* sp.-*F. rumphill*	6	20	15	10	9
*Eupristina altissima*	7	73	16	10	11
*Blastophaga* sp.-*F. abeli*	11	22	20	13	15
*B.* sp.-*F. pyriformis*	11	20	13	13	10
*B.* sp.-*F. formosa*	9	27	14	14	14
*B.* sp.-*F. erecta* var. *beecheyana*	12	30	14	17	15
*B.* sp.-*F. variolosa*	29	27	24	11	12
*Ceratosolen appendiculatus*	14	78	19	22	19
*C. fusciceps*	10	63	16	18	15
*C. gravelyi*	11	43	12	11	9
*C. solmsi*	9	47	14	14	12
*C. constrictus*	12	34	16	14	6
*Kradibia tentacularis*	35	22	32	14	15
Mean for 25 fig wasps	14.72	33.64	19.48	13.16	10.2
SE	7.45	16.39	6.59	3.56	3.63
*Nasonia vitripennis*	62	474	20	53	143
*Copidosoma floridanum*	47	186	16	38	57
*Apis mellifera*	21	343	13	31	68
*Polistes canadensis*	11	181	18	29	48
*Acromyrmex echinatior*	17	681	31	38	93
*Orussus abietinus*	9	94	26	31	39
*Drosophila melanogaster*	48	129	9	60	82
Mean for seven other insects	30.72	298.29	19	40	75.71
SE	21.16	214.51	7.53	11.97	35.10

## Data Availability

The raw sequence data are available in the Genome Sequence Archive (GSA) at the National Genomics Data Center, Chinese Academy of Sciences (https://bigd.big.ac.cn/gsa; accessed on 23 March 2001), under accession number PRJCA004756.

## Supplementary Materials

The following are available online at https://www.mdpi.com/article/10.3390/insects12090815/s1. Figure S1: Phylogenetic relationships with rapidly evolving gene (REG) clusters and positively selected gene (PSG) clusters of 25 fig wasp species and 7 other insect species: (a) comparison of the number of REGs and PSGs among 25 fig wasp species; (b) phylogenetic tree with the numbers of REGs and PSGs mapped on to internal branches and outer species, Table S1: Summary of assembly results of transcriptome data for 25 fig wasp species, Table S2: Summary of functional annotation of unigenes of 25 fig wasp species, Excel S1: Comparison of the numbers of gene families in five insect species between genomic data and transcriptome data, Excel S2: Gene family enriched in GO and KEGG for Agaonidae and four genus clades, Excel S3: Gene family enriched in GO for 25 fig wasp species, Excel S4: Gene family enriched in KEGG pathways for 25 fig wasp species, Excel S5: Rapidly evolving genes (REGs) enriched in GO for fig wasps at the levels of species, genus, and Agaonidae, Excel S6: Rapidly evolving genes (REGs) enriched in KEGG for fig wasps at the levels of species, genus, and Agaonidae, Excel S7: Positively selected genes (PSGs) enriched in GO for fig wasps at the levels of species, genus, and Agaonidae, Excel S8: Positively selected genes (PSGs) enriched in KEGG for fig wasps at the levels of species, genus, and Agaonidae, Excel S9: Positively selected genes (PSGs) for fig wasps at the levels of species, genus, and Agaonidae.
