# Supplementary material for: Adaptation of Fig Wasps (Agaodinae) to Their Host Revealed by Large-Scale Transcriptomic Data
Source: Insects. 2021 Sep 11;12(9):815. doi: 10.3390/insects12090815 (PMC8471397; doi:10.3390/insects12090815)
Supplement: Supplementary file 1 [file insects-12-00815-s001.zip › supplementary data/Table S1-S2.pdf]

Table S1. Summary of assembly results of transcriptome data for 25 fig wasp species

Species	Raw reads (M)	Clean reads (M)	Total number of transcripts	Total number of coding transcripts	Total number of unigenes	Coding unigenes		
						Total number	N50 length (bp)	GC%
Platyscapa. quadraticeps	23.71	22.4	46,136	29,486	18,968	14,792	1958	36.52%
P. sp.- *F. concinna*	25.22	24.08	36,172	23,430	12,933	9,764	2728	36.69%
P. sp. - *F. rumphii*	22.72	21.46	39,384	23,481	13,935	9,618	2409	37.99%
Eupristina altissima	27.30	26.21	44,258	27,064	16,368	11,400	2477	37.45%
Valisia javana sp. 1	21.32	20.09	36,024	22,468	16,239	10,295	2701	37.68%
V. javana sp. 2	20.78	19.58	48,063	33,129	26,637	20,735	1713	37.38%
V. javana sp. 7	21.96	21.06	42,850	26,714	21,355	14,528	2061	36.97%
V. javana sp. 8	23.78	22.54	42,058	25,008	16,876	10,895	2539	36.86%
V. esquirolianae	26.80	25.71	45,285	27,557	17,421	12079	2653	37.21%
V. medusa	20.26	19.32	59,773	33,883	19,692	12,373	2042	36.77%
V. cf *filippina*	25.71	22.54	183,404	75,706	139,669	59,860	774	47.10%
V. malayana	22.53	21.31	43,592	25,733	13,955	9,579	2345	36.11%
V. compacta	21.32	20.09	62,652	33,460	22,632	13,739	1947	36.64%
V. sp.- *F. langkokensis*	26.81	25.65	49,115	28,856	15,206	10,427	2546	36.75%
B. sp.-*F. abeli*	26.42	25.73	67,309	39,756	19,412	13,801	1965	40.01%
B. sp.-*F. pyriformis*	24.85	24.13	58,305	34,306	17,491	11,803	2108	39.43%
B. sp.-*F. erecta var. beecheyana*	30.62	29.58	59,345	34,685	17,357	11,839	2285	37.52%
B. sp.-*F. formosa*	27.12	26.35	74,714	41,309	20,222	13,200	2297	39.20%
B. sp.-*F. variolosa*	25.71	24.84	71,482	44,197	24,438	18,159	2073	40.35%
Ceratosolen appendiculatus	25.44	24.5	58,303	37,243	27,749	19,692	1875	44.91%

C. fusciceps	20.12	18.93	80,161	38,280	28,912	17,304	1521	40.86%
C. gravelyi	20.13	19.08	42,529	24,183	15,620	10,103	1980	38.12%
C. constrictus	27.76	26.91	82,380	44,976	24,451	15,919	1933	40.27%
C. solmi	28.82	26.87	56,671	30,749	16,518	10,143	2498	38.51%
Kradibia tentacularis	21.75	20.55	69,155	39,696	26,640	17,271	1670	40.58%

Table S2. Summary of functional annotation of unigenes of 25 fig wasp species

Species	Number of Proteins	Rate of Nr annotated	Rate of SwissProt annotated	Rate of KOG annotated	Rate of eggNOG annotated	Rate of PFAM annotated	Rate of KEGG annotated	Rate of GO annotated	Rate of annotated	Number of Proteins annotated
Platyscapa. quadraticeps	14792	79.08%	64.18%	48.13%	76.16%	73.43%	60.96%	51.40%	86.24%	12757
P. sp.- *F. concinna*	9764	85.89%	66.15%	46.89%	82.72%	74.71%	58.47%	52.92%	88.99%	8689
P. sp. - *F. rumphii*	9618	77.66%	60.87%	44.47%	74.79%	67.97%	55.48%	49.14%	81.71%	7859
Eupristina altissima	11400	82.83%	65.15%	45.81%	79.82%	73.12%	57.39%	51.46%	86.64%	9877
Valisia javana sp. 1	10295	79.24%	60.97%	43.29%	76.23%	69.14%	53.23%	48.46%	81.76%	8417
V. javana sp. 2	20735	77.32%	62.53%	46.36%	74.06%	72.58%	58.16%	47.20%	84.70%	17563
V. javana sp. 7	14528	80.76%	62.27%	45.09%	76.60%	70.43%	57.50%	49.91%	85.50%	12422
V. javana sp. 8	10895	79.93%	61.62%	43.48%	77.06%	70.19%	54.92%	49.73%	83.49%	9096
V. esquirolianae	12079	76.39%	57.84%	41.20%	72.08%	66.80%	52.67%	45.67%	79.71%	9628
V. medusa	12373	80.54%	61.58%	45.23%	76.51%	70.11%	57.59%	50.13%	85.88%	10626
V. cf *filippina*	59860	75.50%	51.17%	27.18%	61.03%	66.80%	56.10%	33.72%	87.44%	52340
V. malayana	9579	82.95%	64.58%	46.22%	79.91%	72.35%	59.17%	52.06%	87.42%	8374
V. compacta	13739	81.44%	64.17%	46.24%	78.48%	72.01%	59.02%	49.92%	87.09%	11965
V. sp. - *F. langkokensis*	10427	85.11%	66.89%	47.03%	82.11%	74.83%	60.27%	53.24%	89.53%	9335
B. sp.-*F. abeli*	13801	76.69%	57.75%	41.21%	73.97%	66.47%	54.06%	47.32%	82.65%	11407

B. sp.-*F.pyriformis*	11803	78.90%	60.13%	42.92%	76.34%	68.22%	54.95%	49.41%	84.09%	9925
B. sp.-*F. erecta var. beecheyana*	11839	83.97%	65.03%	44.79%	81.15%	72.76%	56.06%	52.35%	88.16%	10437
B. sp.-*F. formosa*	13200	71.45%	54.37%	37.73%	68.13%	61.99%	49.67%	43.28%	75.99%	10031
B. sp.-*F. variolosa*	18159	78.97%	60.59%	44.20%	75.45%	68.70%	57.43%	49.57%	84.56%	15356
Ceratosolen appendiculatus	19692	77.37%	59.72%	44.25%	72.79%	67.81%	39.59%	40.69%	88.48%	17423
C. fusciceps	17304	72.03%	56.69%	40.26%	69.02%	64.78%	54.26%	43.88%	79.56%	13767
C. gravelyi	10103	77.45%	59.38%	43.66%	73.85%	67.12%	56.78%	48.34%	84.58%	8545
C. constrictus	15919	75.12%	60.13%	44.68%	71.93%	68.72%	57.24%	48.67%	81.98%	13050
C. solmi	10143	81.17%	62.27%	43.27%	77.93%	70.01%	55.20%	49.98%	84.91%	8612
Kradibia tentacularis	17271	75.59%	58.08%	43.52%	71.50%	65.54%	55.64%	47.26%	81.38%	14055
Median	12373	78.97%	60.97%	44.25%	76.16%	69.14%	56.1%	49.41%	84.7%	10437
