# Supplementary figures and images for: Adaptation of Fig Wasps (Agaodinae) to Their Host Revealed by Large-Scale Transcriptomic Data
Source: Insects. 2021 Sep 11;12(9):815. doi: 10.3390/insects12090815 (PMC8471397; doi:10.3390/insects12090815)

(a)

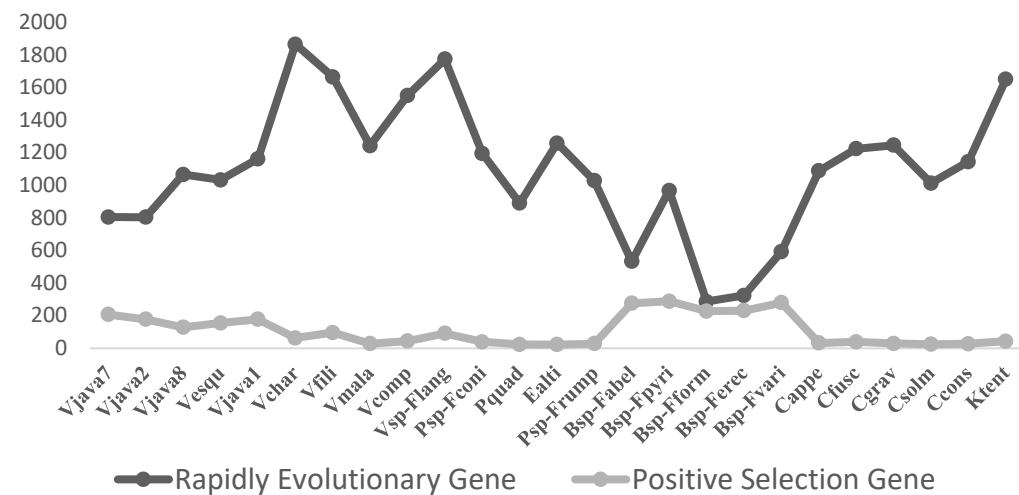

(b)

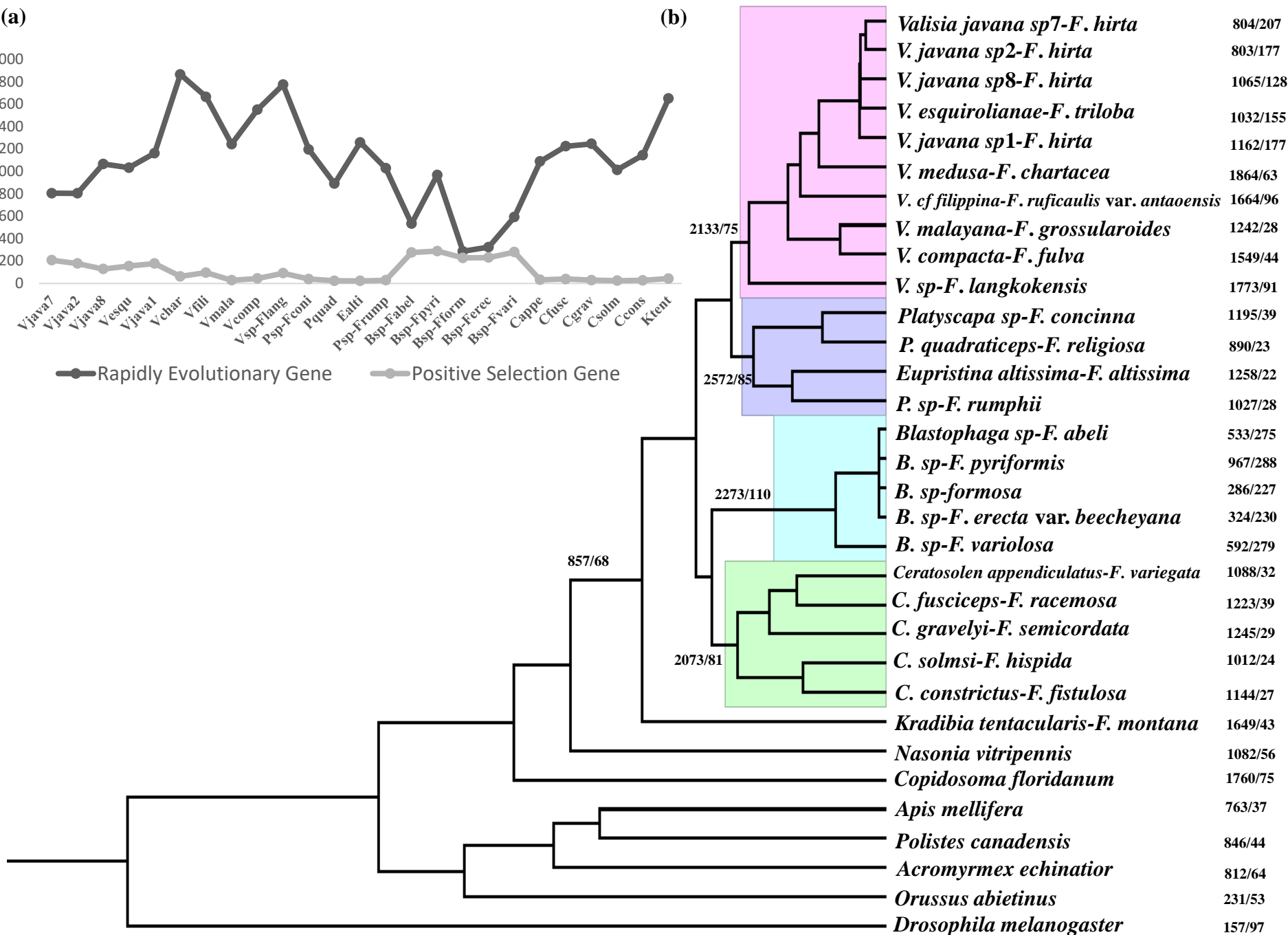

Supplement: Supplementary file 1 [file insects-12-00815-s001.zip › supplementary data/Figure S1.pdf]
